# Predatory bacteria are nontoxic to the rabbit ocular surface

**DOI:** 10.1038/srep30987

**Published:** 2016-08-16

**Authors:** Eric G. Romanowski, Nicholas A. Stella, Kimberly M. Brothers, Kathleen A. Yates, Martha L. Funderburgh, James L. Funderburgh, Shilpi Gupta, Sonal Dharani, Daniel E. Kadouri, Robert M. Q. Shanks

**Affiliations:** 1 University of Pittsburgh, Department of Ophthalmology, Eye & Ear Institute, 203 Lothrop St., Pittsburgh, PA, 15213; 2 Charles T. Campbell Ophthalmic Microbiology Laboratory; 3 Fox Center of Vision Restoration, 203 Lothrop Street, Suite 833, Pittsburgh, PA 15213; 4 Department of Oral Biology, Rutgers School of Dental Medicine, Newark, NJ, USA

## Abstract

Given the increasing emergence of antimicrobial resistant microbes and the near absent development of new antibiotic classes, innovative new therapeutic approaches to address this global problem are necessary. The use of predatory bacteria, bacteria that prey upon other bacteria, is gaining interest as an “out of the box” therapeutic treatment for multidrug resistant pathogenic bacterial infections. Before a new antimicrobial agent is used to treat infections, it must be tested for safety. The goal of this study was to test the tolerability of bacteria on the ocular surface using *in vitro* and *in vivo* models. Predatory bacteria *Bdellovibrio bacteriovorus* and *Micavibrio aeruginosavorus* were found to be non-toxic to human corneal stromal keratocytes *in vitro*; however, they did induce production of the proinflammatory chemokine IL-8 but not IL-1β. Predatory bacteria did not induce inflammation on the ocular surface of rabbit eyes, with and without corneal epithelial abrasions. Unlike a standard of care antibiotic vancomycin, predatory bacteria did not inhibit corneal epithelial wound healing or increase clinical inflammatory signs *in vivo*. Together these data support the safety of predatory bacteria on the ocular surface, but future studies are warranted regarding the use predatory bacteria in deeper tissues of the eye.

Emerging antibiotic resistance by pathogenic bacteria is considered a global threat and is responsible for thousands of deaths and millions of dollars spent on health care each year^1^,^2^,^3^,^4^. With few new antibiotic classes being developed^5^, novel methods to treat bacterial infections are becoming necessary. Alternative methods such as bacteriophage therapy show promise, but can be hampered by narrow host range and rapid development of resistance by bacteria^6^. Like bacteriophage, predatory bacteria, such as *Bdellovibrio bacteriovorus* may be useful as a therapeutic^7^,^8^,^9^. Predatory bacteria generally have a broader host-range than phage^10^,^11^,^12^,^13^, and genetically stable resistance of bacteria to predatory bacteria has yet to be described^14^. Importantly, multidrug resistant bacteria are killed by predatory bacteria, just as readily as their non-resistant kin^15^,^16^.

If predatory bacteria are to be used as therapeutic agents, thorough testing must be performed to demonstrate a lack of toxicity. Previous studies have shown a lack of toxicity of *B. bacteriovorus* and *Micavibrio aeruginosavorus* to a number of cell types *in vitro*^16^,^17^,^18^. Similarly, animal studies supported the safety of predatory bacteria in dosed *per os* with *B. bacteriovorus*^10^ and mice treated intravenously and in the respiratory tract with *B. bacteriovorus* and *M. aeruginosavorus*^19^. Furthermore, *B. bacteriovorus* was found in the gut microflora of healthy humans by genetic analysis^20^.

In addition to the use of predatory bacteria as therapeutic agents in the oral cavity or gut, topical application of these microbes on skin or the ocular surface may be feasible^8^. Whether or not the ocular surface tolerates aggressive application of predatory bacteria has yet to be examined. Additionally, the impact of predatory bacteria on wound healing has not been examined. The goal of this study is to test the safety of *B. bacteriovorus* and *M. aeruginosavorus* for topical application using the eye and ocular cells as models. Ocular clinical signs of inflammation and wound healing were measured. The results of this study are consistent with predatory bacteria being non-toxic to the ocular surface.

## Materials and Methods

### Tissue culture, cytotoxicity analysis, and ELISA

De-identified corneas from organ donors were obtained from the National Disease Research Interchange (Philadelphia, PA) or from Center for Organ Recovery and Education (Pittsburgh, PA). The use of de-identified tissue from non-living individuals is not human subject research under DHHS regulation 45CFR46, and exemption from human subjects regulation was recognized by the Institutional Review Board of the University of Pittsburgh. Ethical aspects of the research protocols were approved by and performed in accordance with guidelines of the Committee for Oversight of Research Involving the Dead. Human stromal keratocytes were derived from adult human corneal stromal stem cells as previously described^21^. Monolayers of stem cells were grown to confluence, transferred to keratocyte differentiation medium (KDM) containing FGF2 and TGF-ß3^22^ for 3 days, then challenged by predatory bacteria, *Pseudomonas aeruginosa*, triton X-100 (0.25%), or lysogeny broth (LB)^23^,^24^ as previously described^16^.

*P. aeruginosa* strain PA14^25^ was grown to stationary phase in LB medium, washed with saline supplemented with glucose (0.1%), and suspended in KDM medium (50 μl). *P. aeruginosa* was added to wells with keratocytes covered with 450 μl of KDM medium, to create a multiplicity of infection (MOI) of 200 bacteria per keratocyte. Predatory bacteria were cultured and purified as previously described^19^. Predatory bacteria were added at an MOI of 594 for *B. bacteriovorus* HD100, 516 for *B. bacteriovorus* 109J, and 161 for *M. aeruginosavorus* ARL-13.

Following 4–24 h exposure to challenges, the cell layers were washed gently with saline with glucose (0.1%) to remove bacteria and suspended in KDM with Presto Blue viability reagent (Life Technologies) with amikacin (40 μg/ml) to prevent growth of remaining bacteria. Presto Blue was used as previously described^26^. KDM medium was used as a control (mock) to determine the maximum viability value, and Triton X-100 (0.25%) was used to kill the keratocytes to determine the minimum viability value. The experiment was performed four times.

ELISA experiments were performed using supernatants from the above cytotoxicity experiments as previously described^16^. The limit of detection for the CXCL8/IL-8 ELISA kit was 31.2 pg/ml (R&D Systems) and 2 pg/ml for TNFα (ThermoFisher). Each experiment was performed twice with independent samples.

### Ocular tolerability and wound healing experiments

New Zealand White (NZW) female rabbits weighing 1.1–1.4 kg were purchased from Charles River (Wilmington, MA, USA). Rabbits were housed under specific pathogen-free conditions at the University of Pittsburgh animal facility. Animal use protocols were approved by the Institutional Animal Care and Use Committee of the University of Pittsburgh (Protocol #15025331) and the Animal Care and Use Review Office of the US. Army Medical Research and Material Command, and were performed in accordance with the approved guidelines. All studies conformed to the ARVO Statement for the Use of Animals in Ophthalmic and Vision Research.

Following general anesthesia with 40 mg/kg ketamine (Ketathesia, Henry Schein Animal Health, Dublin OH) and 4 mg/kg xylazine (AnaSed Injection, Lloyd Laboratories, Shenandoah, IA) administered intramuscularly and topical anesthesia with two drops of 0.5% proparacaine (Proparacaine Hydrochloride Ophthalmic Solution, USP, Sandoz, Princeton, NJ), epithelial defects were introduced onto corneas on the right eyes of the rabbits using an Amoils epithelial scrubber (Innovative Excimer Solutions, Inc, Toronto, ON, Canada) with a sterile 6.5 mm brush, which makes a ~7 mm abrasion. The rabbits were immediately treated with analgesia in the form of intramuscular injections of 1.5 mg/kg of ketoprofen (Ketofen, Zoetis Inc., Kalamazoo, MI). Nothing was done to the left eyes.

The rabbits were then divided into 5 groups, (n = 3 per group): (1) Saline; (2) Vancomycin (5% w/v); (3) *Micavibrio aeruginosavorus* strain ARL-13 (Mica); (4) *Bdellovibrio bacteriovorus* strain HD100 (BD HD100); (5) *Bdellovibrio bacteriovorus* strain 109J (BD 109J). Ocular clinical signs were obtained using a slit lamp and evaluated following a modified MacDonald-Shadduck scoring system^27^ by a skilled observer (EGR). The scoring system has a maximum score of 26, and evaluates corneal opacity (up to 6 points), area of corneal opacity (up to 4 points), corneal vascularization (up to 2 points), conjunctival congestion (up to 3 points), conjunctival chemosis and swelling (up to 4 points), conjunctival discharge (up to 3 points), and area of cornea staining with topical fluorescein (FLUORESOFT-0.35%, Alden Optical, Lancaster, NY), a dye that reveals defects in the corneal epithelium when illuminated with a cobalt blue light (up to 4 points). Both eyes of each rabbit were dosed with one 50 μl drop of predatory bacteria, saline, or vancomycin at 1 hour intervals, 5 times per day, for five days, and were evaluated on the first day before treatment (day 0), and each day after treatment. Drops contained either 1.30 × 10^7^ PFU of *M. aeruginosavorus*, or 4.80 × 10^7^ PFU of *B. bacteriovorus* HD100, or 4.13 × 10^7^ PFU of *B. bacteriovorus* 109J. Evaluations continued at intervals over an additional week.

The study was repeated on three occasions, with one animal removed from the saline group due to injury incurred during shipping. For two experiments, the largest diameters of the fluorescein stained epithelial defects were measured using a ruler and the slit lamp to a resolution of 0.5 mm.

Topical vancomycin 5% was prepared by reconstituting 500 mg of vancomycin hydrochloride (Fresenius Kabl USA LLC, Lake Zurich, IL) in 10 ml of sterile water. Pharmaceutical grade saline (0.9% Sodium Chloride Injection USP, Baxter Healthcare Corp. Deerfield, IL) was purchased from the inpatient pharmacy at the University of Pittsburgh Medical Center. The evaluations on day 1 and 3 were omitted for one iteration of the experiment.

### Statistical analysis

Kruskal-Wallis with Dunn’s multicomparison and two-tailed Fisher Exact tests were performed with Graphpad Prism software, and two-tailed Monte Carlo randomization test with 1000 shuffles was performed with True Epistat software. Significance was set at p < 0.05.

## Results

### Susceptibility of human stromal keratocytes to predatory bacteria

**Figure 1:**
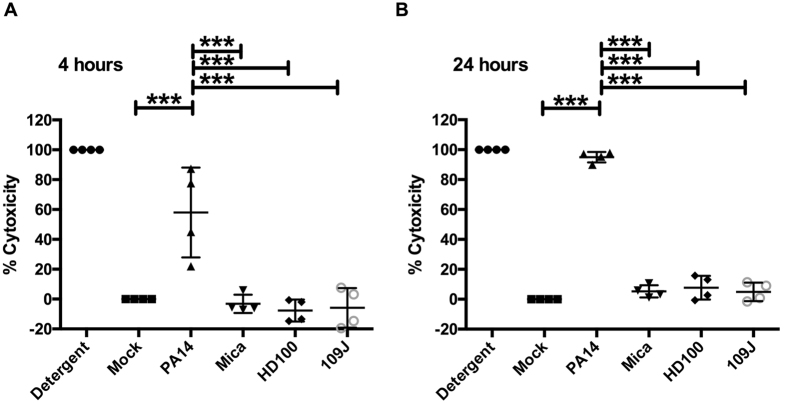
Predatory bacteria are non-cytotoxic to human keratocytes *in vitro*. Mean and standard deviations from four independent experiments are shown. Asterisks indicate significant differences using ANOVA with Tukey’s post-hoc test (***p < 0.001). There was no difference in cytotoxicity between Mock and predatory bacteria. PA14 indicates *P. aeruginosa* strain PA14, Mica = *M. aeruginosavorus*, HD100 and 109J indicate *B. bacteriovorus* strains. (**A**) 4 h, (**B**) 24 h.

Stromal keratocytes play an important role in wound healing and in maintenance of corneal clarity, which is required for vision^28^. Here we tested whether predatory bacteria were cytotoxic to monolayers of human keratocytes *in vitro* as a primary step in determining their safety for ocular use. Monolayers were challenged for 4 and 24 hours with predatory bacteria. Compared to the mock control and positive controls for cytotoxicity, detergent and *P. aeruginosa* PA14, a cytotoxic strain, were significantly more damaging to keratocytes than the PBS negative control and predatory bacteria at both 4 and 24 h (p < 0.001, ANOVA with Tukey’s post-test) (Fig. 1A,B). Predatory bacteria were not significantly different than the mock treatment group (PBS) for cytotoxicity (p > 0.5, ANOVA with Tukey’s post-test), (Fig. 1A,B).

**Figure 2:**
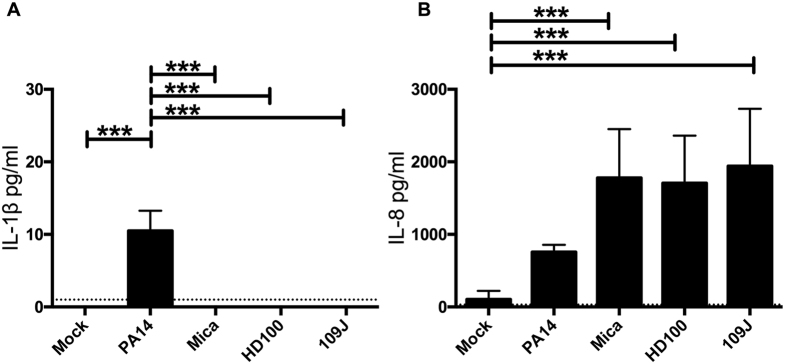
Predatory bacteria induce an IL-8, but not IL-1β response from corneal stromal keratocytes *in vitro*. Pro-inflammatory cytokines IL-1β (**A**) and IL-8 (**B**) were measured by ELISA. Cell supernatants taken from keratocytes after 4 hours of incubation with medium only negative control (Mock), positive control *Pseudomonas aeruginosa* strain PA14 (MOI = 200), and experimental strains *M. aeruginosavorus* (Mica, MOI = 161), *B. bacteriovorus* strain HD100 (MOI = 594), and *B. bacteriovorus* strain 109J (MOI = 516). The average and standard deviation from n = 7 independent data points from 2 experiments are shown. Asterisks indicate significant differences (*p < 0.05, **p < 0.01, ***p < 0.001 determined using ANOVA with Tukey’s post-test). Limits of assay detection are denoted by the dotted horizontal lines.

Pro-inflammatory cytokines IL-1β and IL-8 were measured from the human stromal keratocyte supernatants after exposure to *P. aeruginosa* and predatory bacteria by ELISA. After 4 hours of exposure to *P. aeruginosa* there was a significant increase in IL-1 β, but not IL-8 levels in the supernatants of human keratocytes (Fig. 2A). Predatory bacteria did not induce IL-1β, but did induce IL-8 levels (Fig. 2B).

### *In vivo* ocular tolerability of predatory bacteria

Since the predatory bacteria were non-cytotoxic to ocular epithelial cells^16^ and keratocytes (Fig. 1A,B), their safety in an animal model was tested.

**Figure 3:**
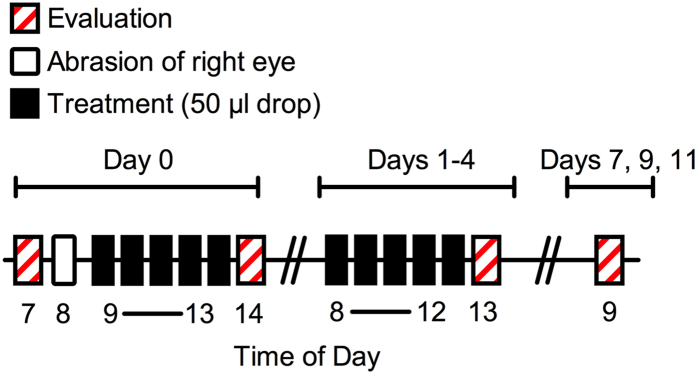
Time line and experimental outline for *in vivo* ocular tolerability experiments. The corneas of NZW rabbits were abraded (right eye) or left intact (left eye), and exposed to drops of saline (negative control for toxicity), vancomycin (positive control for toxicity), or predatory bacteria (>10^7^ PFU per drop). Drops were administered at 1 hour intervals, five times per day for the first 5 days. Each day the eyes were treated with fluorescein and evaluated for clinical signs of inflammation and toxicity using a MacDonald-Shadduck scoring system. The diameter of the corneal defect on the right eye was measured with a ruler.

**Figure 4:**
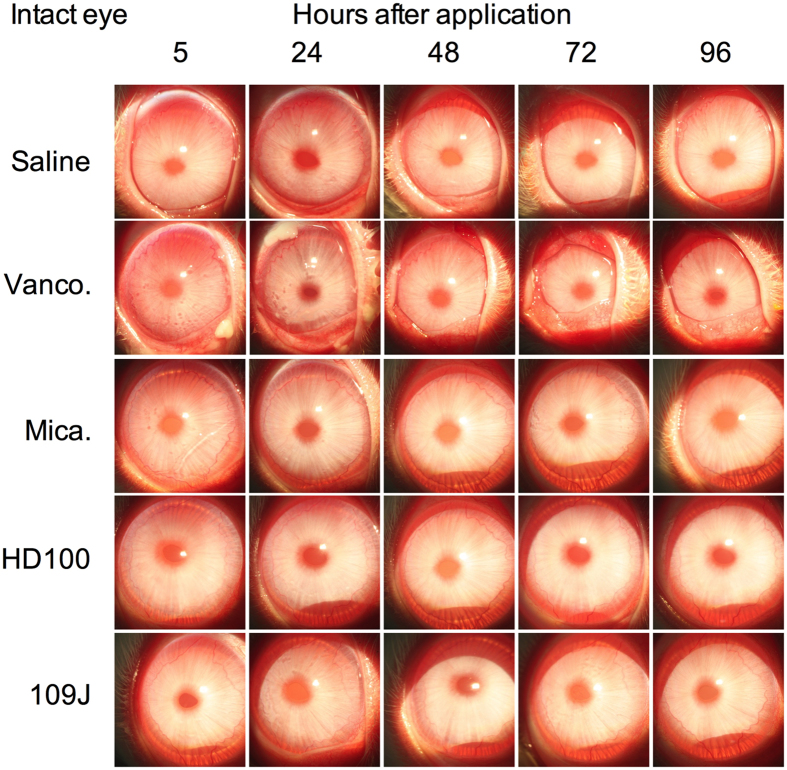
Predatory bacteria do not cause ocular toxicity in the intact (left) of rabbits. Eyes of NZW rabbits were photographed using a slit-lamp camera at time points indicated above. Inflammatory signs are observed in the vancomycin treatment group, but not the other groups. The eye of a representative rabbit for each group is shown.

Corneas on the left eyes of New Zealand White Rabbits were exposed to predatory bacteria (≥10^7^ PFU) five times a day for five days and evaluated for an additional week (Fig. 3). Eyes were stained with fluorescein and graded for ocular clinical signs of inflammation using a slit-lamp and quantified according to a modified MacDonald-Shadduck scoring system. Vancomycin (5%) was used as a positive control for ocular surface toxicity. Compared to the saline negative control, vancomycin was clearly more toxic to the ocular surface, inducing inflammation and swelling (chemosis) of the conjunctiva and nictitating membrane along with the production of ocular mucous (Fig. 4). Eyes exposed to each of the predatory bacteria were indistinguishable from the saline treatment group (Fig. 4).

**Figure 5:**
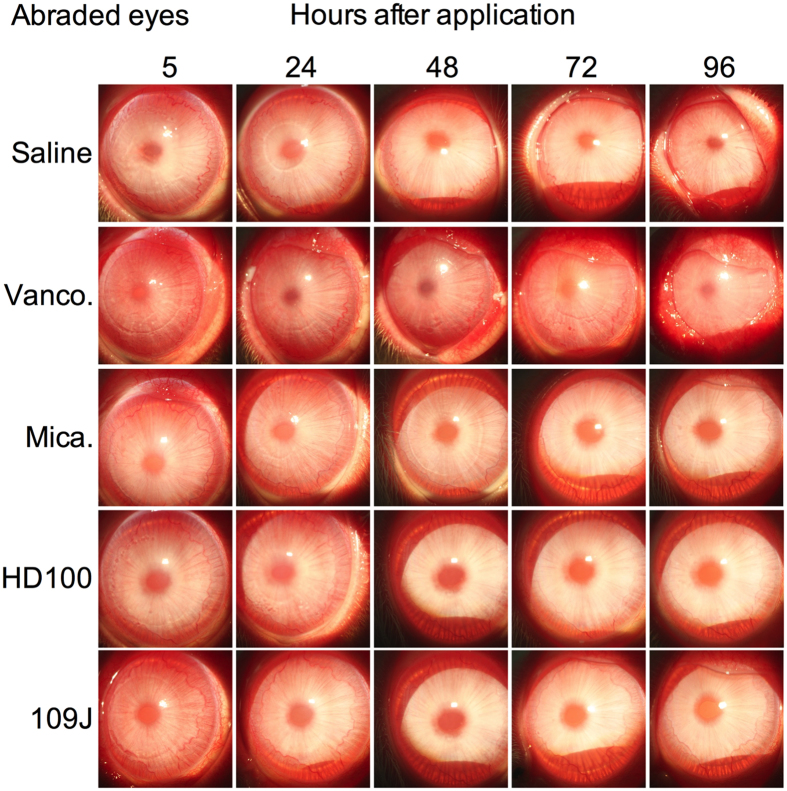
Predatory bacteria do not cause ocular toxicity in abraded (right) rabbit eyes. A 7 mm diameter region of the cornea of NZW rabbits was abraded of epithelial cells. Subsequently, eyes were treated with saline, vancomycin, and predatory bacteria, and photographed with a slit-lamp camera. Clear inflammatory signs are observed in all groups at the 5 hour time point, that persisted only in the vancomycin treatment group. The eye of a representative rabbit for each group is shown.

**Figure 6:**
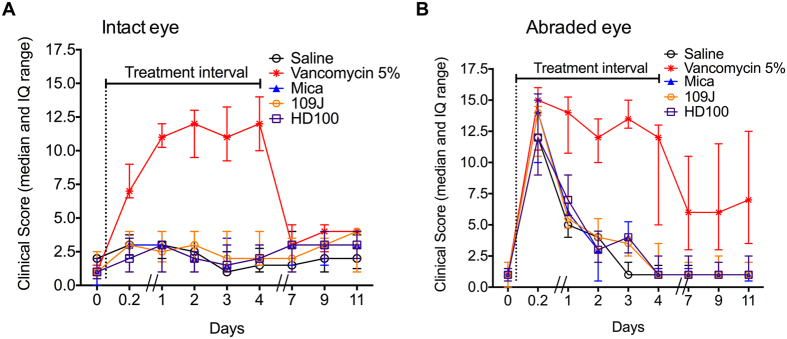
Clinical signs of rabbit eyes exposed to predatory bacteria were similar to those exposed to saline. The intact eye (**A**) and abraded eye (**B**) of NZW rabbits were treated with saline, vancomycin, and predatory bacteria 5 times a day for 5 days (treatment interval). Eyes were stained with fluorescein and graded using a modified MacDonald-Shadduck grading system. The median and interquartile range (IQ) are shown (n ≥ 8 animals per group).

Because microbial keratitis is generally associated with erosion of the corneal epithelium and wounded or inflamed eyes can be more sensitive to topical therapeutic approaches, the above *in vivo* tolerability study was also performed using eyes with epithelial defects. An epithelial scrubber, used by ophthalmologists on human eyes, was used to produce 7 mm diameter zones free of corneal epithelium in the right eyes of the above rabbits (abraded eye). As expected, the baseline clinical scores were higher in the saline treatment of the wounded eyes than the non-wounded eyes for the first several days; afterwards there was no quantitative difference (Fig. 5). Vancomycin caused clear toxicity, in some cases for the entire 12 day course of the experiment. Predatory bacteria mirrored the saline treatment group, suggesting that they are well tolerated even by eyes with damaged surface epithelia in both intact and abraded eyes (Figs 5 and 6).

Statistical analysis (Kruskal-Wallis analysis with Dunn’s multicomparision test) of combined clinical scores for individual time points indicate no statistical difference between the saline and other groups of the intact left eyes before treatment (p = 0.63). However, there was a statistical difference between saline and vancomycin (p < 0.001) but not saline and predatory bacteria groups (t = 5 hrs) (p > 0.05).

Similar statistical analysis of total clinical scores for the abraded right eyes indicate no statistical difference between the saline and other groups before treatment (p = 0.91), or just after abrasion and initial treatment (t = 0.2) (p = 0.45). At all subsequent evaluation days (1–11) there was no significant difference between saline and the predatory bacteria, but there was a significant difference between the saline group and the vancomycin group (p < 0.01). The significant difference between the vancomycin and saline groups continued even after treatment was finished (p < 0.01), see days 7–11.

### Impact of predatory bacteria on corneal wound healing

**Figure 7:**
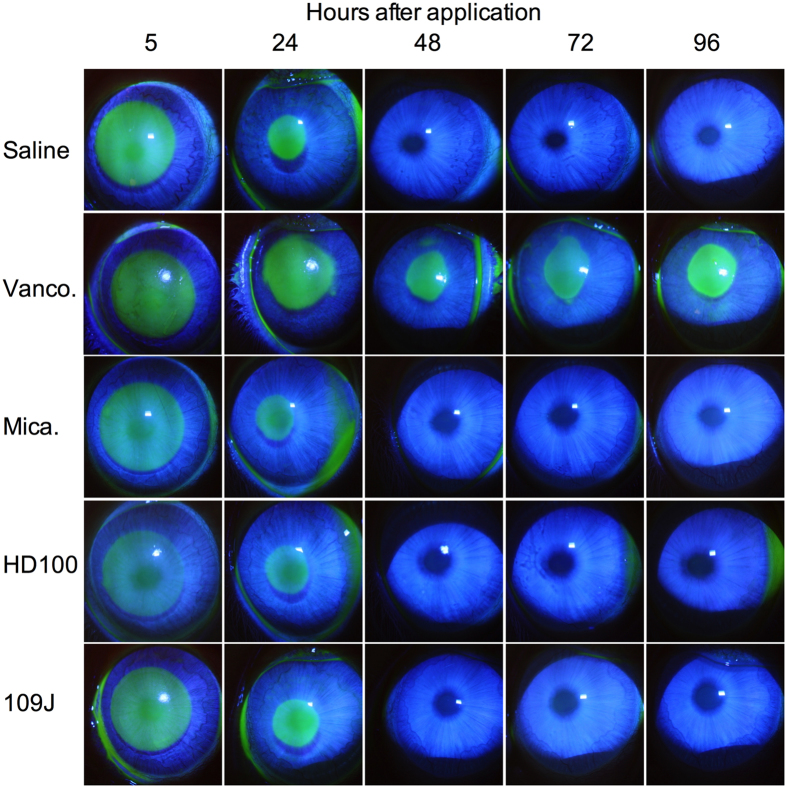
Vancomycin, but not predatory bacteria, inhibited corneal wound healing *in vivo*. A 7 mm diameter region of the cornea of NZW rabbits was abraded of epithelial cells. Eyes were treated with saline, vancomycin, and predatory bacteria. Fluorescein was used to reveal epithelial defects, and eyes were photographed with a slit-lamp camera. The eye of a representative rabbit for each group is shown.

**Figure 8:**
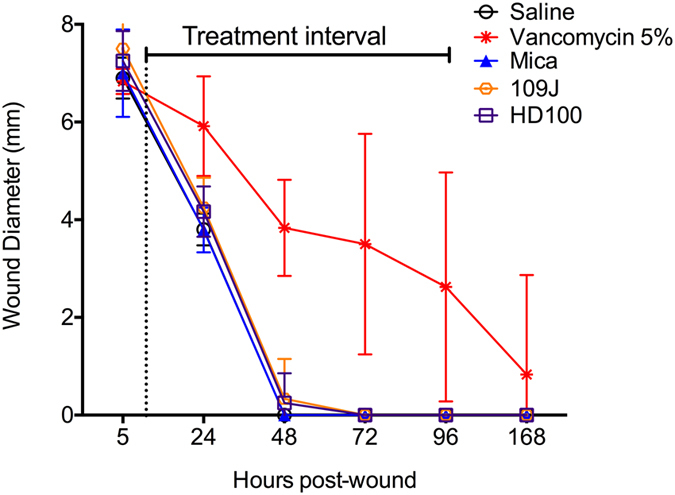
Predatory bacteria do not inhibit corneal wound healing *in vivo*. NZW rabbits with epithelial defects were treated with saline, vancomycin, and predatory bacteria five times a day for five days. Eyes were stained with fluorescein and graded using a modified MacDonald-Shadduck grading system. The average and standard deviation are shown (n = 5 rabbits for the saline group, and 6 for the remaining groups).

Wound healing following infection or trauma is essential for tissue function and to prevent infection. It is generally thought that bacteria play an important role in preventing wound healing associated with chronic wounds, and experimental studies support this hypothesis^29^,^30^,^31^. The epithelial defect, in the right eyes of the above noted rabbits, was used to evaluate whether predatory bacteria inhibit corneal wound healing. Wound closure of the Amoils scrubber treated corneas described above was recorded. With the saline treatment group, the ~7 mm wound was closed by the 48 hour post-wounding evaluation (Fig. 7). Vancomycin, an antibiotic used to treat microbial keratitis caused by methicillin-resistant *Staphylococcus aureus*, strongly inhibited corneal epithelial wound healing. Wound healing by predatory bacteria treated eyes closely mimicked the healing pattern of the saline treatment group (Fig. 8).

When eyes were grouped into healed versus not healed epithelial defect categories, the differences were quite striking. All of the saline and *M. aeruginosavorus* treated epithelial defects healed by 48 h, 5 out of the 6 eyes treated with each *Bdellovibrio* strain had completely healed, and 0 out of 6 of the vancomycin eyes were healed. By 72 h, all of the predatory bacteria treated epithelial defects had completely healed, whereas only one of the vancomycin treated defects had healed by 96 h. Monte Carlo analysis of healed versus non-healed eyes at 48 hours revealed a significant difference (p = 0.003), and Fisher exact tests showed a significant difference between the vancomycin group and all other groups (p < 0.016). At 48 hours, there was no difference between the predatory bacteria groups and the saline group by Fisher Exact test (p = 1).

## Discussion

This study expanded upon a previous *in vitro* study designed to examine whether predatory bacteria could be safely used as an alternative antimicrobial therapy for ocular surface infections^16^. Here, we used cells from a deeper layer of the cornea that would not normally experience microbial assault. Stromal keratocytes were unperturbed by predatory bacteria in cytotoxicity assays lasting up to 24 hours, whereas *P. aeruginosa* caused significant reduction in ocular cell viability. This result supports our previous finding in which predatory bacteria were found to be non-toxic to human limbal corneal epithelial (HCLE) cells^16^,^32^, thus completely different cell type, keratocytes versus epithelial cells, retains their viability when exposed to high MOI of predatory bacteria.

It was suggested that predatory bacteria might bring about a reduced inflammatory response compared to other Gram-negative bacteria. This is attributed to the unusual structure of the lipid A portion of their lipopolysaccharide, which does not interact with TLR4^33^, as well as their membrane encased flagella^34^, which should not interact with TLR5. Unlike a previous study wherein proinflammatory cytokines were not induced from corneal epithelial cells exposed to predatory bacteria^16^, in this study IL-8 was induced from corneal stromal keratocytes by the tested predatory bacteria. We also observed relatively mild induction of IL-8 by *P. aeruginosa* relative to the predatory bacteria; this may be as a result of *P. aeruginosa* killing the keratocytes before the cells could mount a full immune response. Unlike IL-8, predatory bacteria did not induce IL-1β production in exposed corneal stromal keratocytes cells, supporting the non-inflammatory attributes of the predators. In a recent study, mice were exposed to high concentrations of the predatory bacteria, *B. bacteriovorus* 109J, HD100 and *M. aeruginosavorus,* which were delivered via inhalation and direct tail vein injection. It was reported that although inflammatory responses were detected at early time points of 1–3 hours after exposure, the inflammatory effects were not sustainable and were found to return to baseline levels 18 hours after exposure^19^. Furthermore, histological analysis showed no pathological changes in tissue following exposure to the predators, thus it was concluded that any inflammatory response caused by the predator did not impact animal well being or cause tissue inflammation^19^.

Predatory bacteria were well tolerated on the rabbit ocular surface *in vivo* regardless of whether the corneal epithelium was present or removed. This is especially important because wounded eyes can display highly elevated inflammatory responses to certain therapeutics such as antimicrobial peptides^35^. Furthermore, predatory bacteria appeared to have no impact upon corneal wound healing, unlike other bacteria such as *P. aeruginosa* and *Serratia marcescens* that can robustly inhibit corneal epithelial wound healing *in vitro* and *ex vivo*^31^,^36^. The lack of ocular surface toxicity caused by predatory bacteria is especially clear given the toxicity of vancomycin (5%), which is used by clinicians to treat ocular infections. Although, some clinicians are now using a lower concentration of vancomycin (2.5%) to treat ocular infections.

There has been mixed reports on the effect of topical vancomycin on corneal wound healing. Petroutsos and colleagues demonstrated that vancomycin (3.1%) as well as kanamycin and penicillin inhibited corneal wound healing *in vivo* using a rabbit model^37^. In contrast, Lin and Boehnke used an *ex vivo* pig corneal model, and reported that amphotericin B and gentamicin both inhibited corneal wound healing, but vancomycin (5%) did not^38^. The differences between the outcomes of these studies may be due to the use of different organisms, the intact immune system in the *in vivo* model, or the different dosing regimens (3 doses for the Lin study, versus 6 doses per day for four days). Our study continued longer than the previous studies and demonstrated that even after topical vancomycin treatment had ended for several days, the epithelial defects of some of the eyes failed to resolve. The impact on wound healing should be considered with fortified vancomycin, especially when this antibiotic is used for prophylaxis as is commonly done for patients with keratoprostheses^39^.

In summary, this study demonstrates that predatory bacteria are not toxic to ocular cells *in vitro*, are well tolerated on the ocular surface *in vivo*, and do not prohibit corneal epithelial wound healing, and adds to the growing body of studies supporting that predatory bacteria are non-toxic to mammalian tissues.

## Additional Information

**How to cite this article**: Romanowski, E. G. *et al*. Predatory bacteria are nontoxic to the rabbit ocular surface. *Sci. Rep.* **6**, 30987; doi: 10.1038/srep30987 (2016).
